# Retinitis pigmentosa sine pigmento masqueraded as myopia: A case report (CARE)

**DOI:** 10.1097/MD.0000000000024006

**Published:** 2021-01-22

**Authors:** Yi Lu, Xiaodong Sun

**Affiliations:** a Department of Ophthalmology, Shanghai General Hospital, Shanghai JiaoTong University; b Shanghai Key Laboratory of Ocular Fundus Disease; c Shanghai Engineering Center for Visual Science and Photomedicine; d National Clinical Research Center for Eye Diseases; e Shanghai Engineering Center of Precise Diagnosis and Treatment of Eye Diseases, Shanghai, China.

**Keywords:** case report, multimodal imaging, myopia, retinitis pigmentosa sine pigmento, RP1L1

## Abstract

**Introduction:**

Retinitis pigmentosa is a major cause of visual disability and blindness. Photopsia is usually presented in patients with retinal traction caused by posterior vitreous detachment in clinic, which would occur more commonly in those suffer from moderate or high myopia. We describe a patient with leopard-like retinopathy initially complaining of photopsia caused not by myopia but by retinitis pigmentosa.

**Patient concerns:**

A 39-year-old woman with a history of moderate myopia presented to us complaining of photopsia for several days.

**Diagnosis:**

Fundus examination revealed leopard-like retinopathy with normal optic disc and macula appearance in both eyes. The atrophy of retinal pigment epithelium was found in peripheral retina while no bone spicule was present. Retinal multimodal imaging helped in the correct diagnosis of retinitis pigmentosa (sine pigmento), later confirmed by genetic testing.

**Interventions:**

At current no specific treatment was applied, but the patient was required for follow-up observation every six months.

**Outcomes:**

Follow-up observation

**Conclusion:**

This case highlights the potential for retinitis pigmentosa sine pigmento to present with photopsia under cover of myopia and the importance of performing multimodal imaging including fundus autofluorescence for fundus disorders. Careful history review and multimodal imaging with genetic testing would help for the correct diagnosis of retinitis pigmentosa sine pigmento.

## 1 Introduction

Retinitis pigmentosa (RP) is a major cause of visual disability and blindness, affecting more than 1.5 million patients worldwide. RP is the most common inherited retinal dystrophy, characterized by the primary degeneration of rod photoreceptors, then the loss of cone photoreceptors. The initial symptom is reduced night vision, which is followed by a progressive loss of the visual field in a concentric pattern. Fundus abnormalities typically include bone spicule pigmentation predominantly in the periphery and/or mid-periphery, attenuation of retinal vessels, and a waxy pallor of the optic nerve head.^[1]^ But in RP sine pigmento, an atypical form of RP, fundus examination may be unremarkable because the characteristic pigmented epitheliopathy is not seen, and the diagnosis may be missed, particularly during the early stages of the disease, when other features suggestive of RP are usually not yet present (e.g., arterial narrowing, cystoid macular edema, pale disc, and vitreous changes).^[2]^ Photopsia is usually presented in patients with retinal traction caused by posterior vitreous detachment (PVD) in clinic, which would occur more commonly in those suffer from moderate or high myopia. We describe a patient with leopard-like retinopathy initially complaining of photopsia caused not by myopia but by retinitis pigmentosa.

## 2 Case report

**Figure 1:**
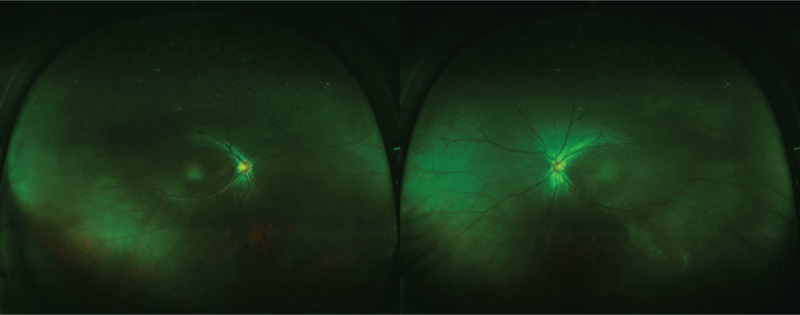
Wild-field retinography OU is unremarkable except for leopard-like retinopathy and mild arterial narrowing.

**Figure 2:**
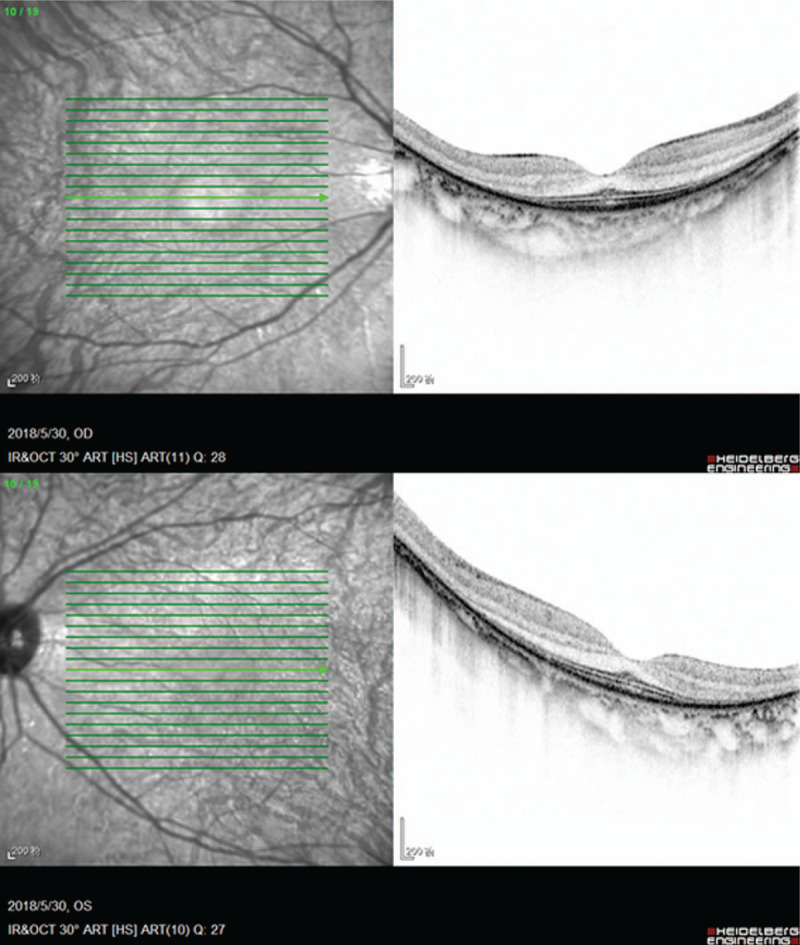
OCT scans show marked disruption of photoreceptor layer outside the macula in OU.

**Figure 3:**
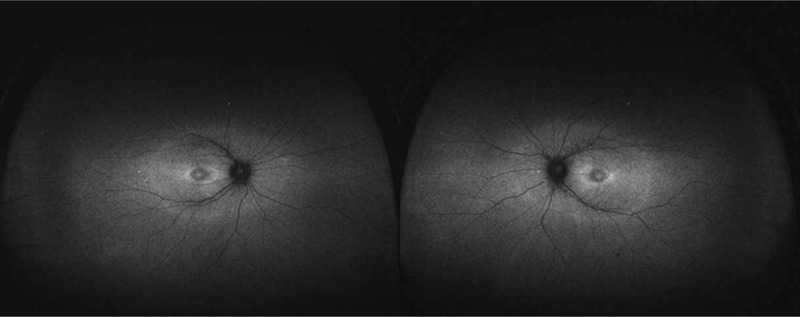
FAF images show an abnormal foveal ring of increased autofluorescence at the posterior pole in OU. FAF = fundus autofluorescence.

**Figure 4:**
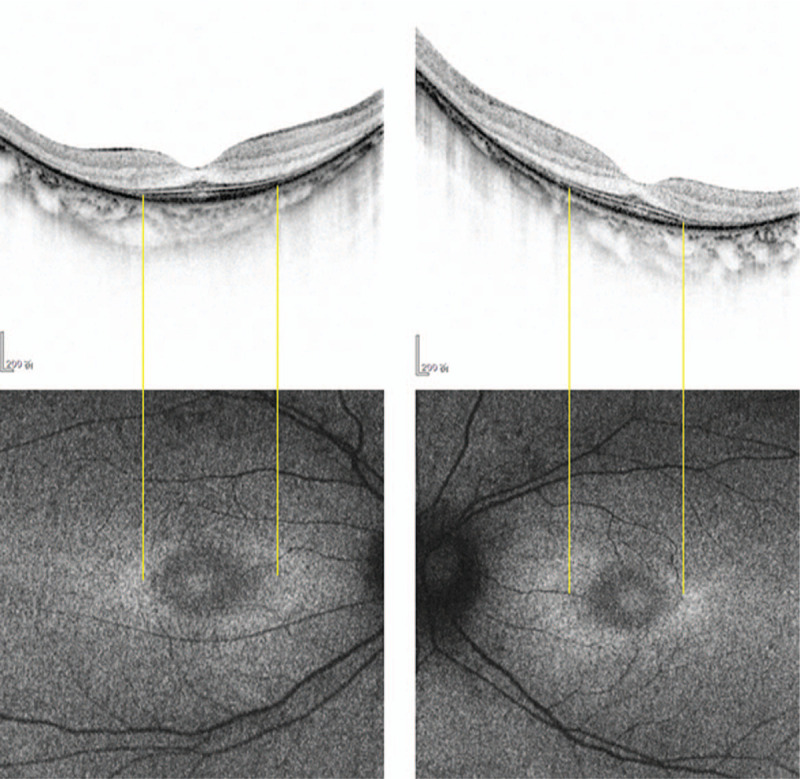
Horizontal OCT (top panel) and FAF images of both eyes of this RP patient. The OCT images show the perifoveal loss of the outer retinal layers. The central preservation of the ellipsoid zone corresponds to the internal edges of the hyperautofluorescent ring visible on FAF. FAF = fundus autofluorescence.

**Figure 5:**
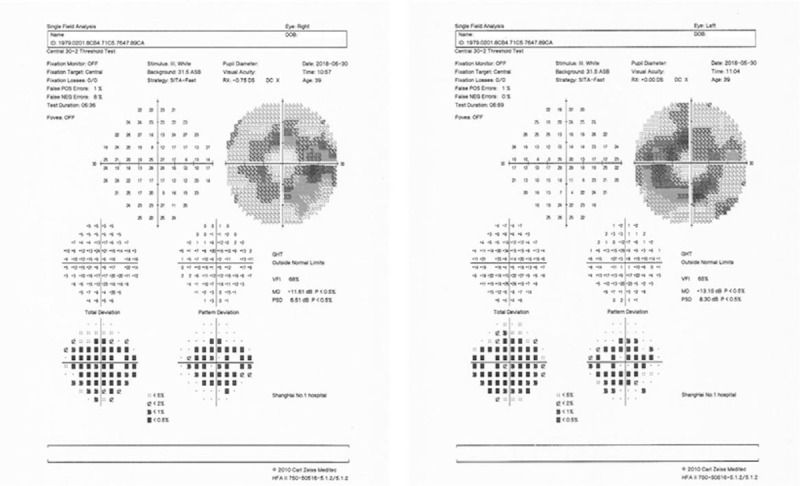
Computerized 30-2 visual field shows symmetrical complete ring scotomas in OU.

**Figure 6:**
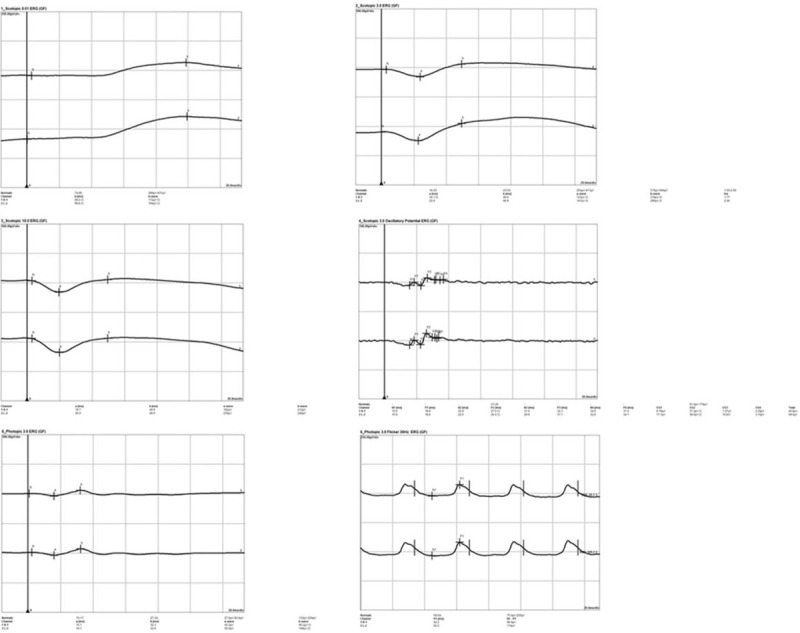
ERG recordings present decreases in the amplitude of responses, and increases in the implicit time, indicating early stage of RP. ERG = electroretinography.

A 39-year-old woman with a history of moderate myopia presented to us complaining of photopsia for several days. Her best-corrected visual acuity was 20/20 in both eyes (right eye: -5.00DS/-0.75DC × 170; left eye: -3.75DS), slit-lamp examination showed normal anterior segment and PVD. The intraocular pressure was 17.1 (right eye) and 17.2 (left eye) mm Hg. Fundus examination (Fig. 1) revealed leopard-like retinopathy with normal optic disc and macula appearance in both eyes. The atrophy of retinal pigment epithelium was found in peripheral retina while no bone spicule was present. Retinal artery vessels were slightly reduced in diameter. At the initial impression, we first considered her photopsia was caused by PVD and the leopard-like retinopathy was related to her myopia. We then conducted optical coherence tomography (OCT) scan to further detect the retinal structure, which showed marked symmetrical disruption of photoreceptor layer outside the macula in both eyes (Fig. 2). From the edge of macula with normal structure to the peripheral area, disorganization of the outer retinal layers progressed gradually, initially at the interdigitation zone, followed by the outer segment of photoreceptors and the ellipsoid zone, then at the myoid zone and the external limiting membrane, and finally accompanied by a decrease in the thickness of the outer nuclear layer. This finding alarmed us for the probability of inherited retinal dystrophy, especially RP. After a thorough history review, the patient admitted suspicious visual field defect from her eighteenth, and occurring of a worse visual performance in dim light condition from four years ago underlying the nyctalopia-related rod dysfunction, as well as a consanguineous marriage of her parents. The patient denied hearing problem. Fundus autofluorescence (FAF) was performed and demonstrated an abnormal foveal ring of increased autofluorescence at the posterior pole with a relatively high level of interocular symmetry (Fig. 3), which was compatible with OCT results. The OCT images showed the perifoveal loss of the outer retinal layers while the central preservation of the ellipsoid zone corresponds to the internal edges of the hyperautofluorescent ring visible on FAF (Fig. 4). The computerized 30-2 visual field test was then performed, which displayed symmetrical complete ring scotomas in both eyes (Fig. 5). The results were compatible with retinitis pigmentosa (sine pigmento) confirmed later by electroretinography studies (indicating early RP) (Fig. 6). Genetic testing was positive for mutation in RP1L1 gene (Table 1), which usually causes occult macular dysfunction but also autosomal recessive retinitis pigmentosa. Considering no treatment is acknowledged as the golden therapy for RP^[3]^ and the patient was currently during the early stage of the disease, no specific treatment was applied, but the patient was required for follow-up observation every six months.

**Table 1:** Genetic test.

Gene	Chr.	Exon	Nucleotide variation	Amino acid change	dbSNP	Hetero-zygosity	Inheritance mode	Variation type
RP1L1NM_178857	8p23.1	4	c.A3971G	p.E1324G	rs4240659	Het	AR	Likely pathogenic
RP1L1NM_178857	8p23.1	4	c.4018_4019ins49bp	p.E1340delins	-	Het	AR	Likely pathogenic

## 3 Discussion

RP is a heterogeneous group of progressive inherited retinal diseases characterized by a degeneration of photoreceptors with visual dysfunction.^[1]^ Photopsia is a common but often-neglected symptom that can be highly disturbing to these patients.^[4]^ This phenomenon may be caused by a lack of afferent nerve impulses in response to photoreceptor degeneration or spontaneous self-signaling activity as a result of inner retina remodeling.^[5]^ Photopsia can occur in the early stages of RP,^[6]^ but is most striking—and particularly disturbing—in patients with more advanced stages of the disease.^[7]^ The location of photopsias appears to be related to residual photoreceptor function.^[6]^ The characteristic feature that gives the name to RP is pigmented epithelial changes in the mid-periphery of the retina. However, there is a high variability in the clinical appearance of the retina in these patients; when the characteristic peripheral bone-like spicule pigmentary changes are present, as happens most of the times, diagnosis of RP is straightforward. But in RP sine pigmento, fundus examination may be unremarkable because the characteristic pigmented epitheliopathy is not seen, and the diagnosis may be missed, particularly during the early stages of the disease.

In our case, the leopard-like retinopathy without the typical bone-like spicule pigmentary changes first misled us consider the main complaint of photopsia caused by myopia. Then the OCT scan results alarmed us of pathologic lesions with the outer layer of retina. Followed, both visual field test and FAF examination supported our suspect of RP. Here, our case highlights the importance and the advantages of performing general retinal multimodal imaging including FAF, when a genetic retinal disease is suspected. Compared with full-field electroretinography, which is still the gold standard for the diagnosis of many inherited retinal disorders, autofluorescence is faster and more readily available and may give the initial clue to the final diagnosis. Results are easily interpretable, and the test is less invasive.

In FAF imaging, the hyperautofluorescent ring represents a transition zone between abnormal and normal retinal function; thus, function is relatively normal within the ring and absent outside of the ring. The level of autofluorescence immediately outside of the ring is relatively preserved, despite severely impaired retinal function. Moreover, the degeneration of photoreceptor cells outside of the ring is reflected in a loss of the ellipsoid zone and the external limiting membrane, as well as a thinning or absence of the outer nuclear layer in the OCT scan.^[8]^ The autofluorescent ring itself corresponds to an area of outer segment dysgenesis and lipofuscin production, with progressive retinal thinning, usually accompanied by loss of the ellipsoid zone at—or close to—the internal edge of the ring.^[8–10]^ In the majority of patients, the autofluorescence measured inside the ring is quantitatively similar to autofluorescence in a healthy eye.^[11]^ Over time, the diameter of the hyperautofluorescent ring grows smaller; although the rate of this reduction in diameter varies, relatively large rings tend to reduce in size more rapidly than small rings. The inner edge of the constricting ring generally matches the progression of cone system dysfunction; in contrast, the loss of rod sensitivity is more widespread and includes the parafoveal area within the ring.^[12]^ Eventually, the ring may disperse, and this phenomenon is correlated with a widespread loss of sensitivity and visual acuity.^[13,14]^

The genetic testing discovered a heterozygous mutation of RP1L1 gene (rs.4240659, c.A3971G; c.4018_4019ins49 bp, p.E1340delins) in this RP patient. RP1L1 is located on the short arm of chromosome 8 at 8p23.1, with a full length of 100 kb. It encodes RP 1-like1, a 2,400 amino-acid protein of the dipicolin family, which contains two N-terminal regions of picolin. It is a retinaldehyde-specific protein, which is conjugated with RP1 and plays a key role in photosensitivity and photoreceptor formation. RP1L1 is reported to be related to occult macular dysfunction but is also found to be a potential genetic cause of autosomal recessive RP.^[15,16]^ In this case, two mutations were detected in RP1L1 gene, one of which (rs.4240659, c.A3971G) was included by Clinvar as “uncertain significance” mutation. According to the American College of Medical Genetics and Genomics guidelines, both were determined to be “likely pathogenic” mutations. However, further study is needed to confirm the function of the genetic variation presented here.

In sum, this case highlights the potential for RP sine pigmento to present with photopsia under cover of myopia and the importance of performing multimodal imaging including FAF for fundus disorders. Careful history review and multimodal imaging with genetic testing would help for the correct diagnosis of RP sine pigmento.

## Author contributions

Y.L. performed data collection, wrote the manuscript and prepared the table and figures. X.S. edited the manuscript.
